# Human Cytomegalovirus Replication and Infection-Induced Syncytia Formation in Labial, Foreskin, and Fetal Lung Fibroblasts

**DOI:** 10.3390/v13122355

**Published:** 2021-11-24

**Authors:** Alexis Aguiar, Melissa Galinato, Maite’ Bradley Silva, Bryant Toth, Michael A. McVoy, Laura Hertel

**Affiliations:** 1 Department of Pediatrics, University of California San Francisco, Oakland, CA 94609, USA; alexis.aguiar@ucsf.edu (A.A.); maite.bradleysilva@ucsf.edu (M.B.S.); 2 Center for Immunobiology & Vaccine Development, Children’s Hospital Oakland Research Institute, Oakland, CA 94609, USA; mgalinato@chori.org; 3 Craniofacial Center, UCSF Benioff Children’s Hospital Oakland, Oakland, CA 94609, USA; tothbryant@gmail.com; 4 Department of Pediatrics, Virginia Commonwealth University, Richmond, VA 23298, USA; michael.mcvoy@vcuhealth.org

**Keywords:** human cytomegalovirus, fibroblasts, tropism, syncytia, functional genomics

## Abstract

Only a handful of cell types, including fibroblasts, epithelial, and endothelial cells, can support human cytomegalovirus (CMV) replication in vitro, in striking contrast to the situation in vivo. While the susceptibility of epithelial and endothelial cells to CMV infection is strongly modulated by their anatomical site of origin, multiple CMV strains have been successfully isolated and propagated on fibroblasts derived from different organs. As oral mucosal cells are likely involved in CMV acquisition, we sought to evaluate the ability of infant labial fibroblasts to support CMV replication, compared to that of commonly used foreskin and fetal lung fibroblasts. No differences were found in the proportion of cells initiating infection, or in the amounts of viral progeny produced after exposure to the fibroblast-adapted CMV strain AD169 or to the endothelial cell-adapted strain TB40/E. Syncytia formation was, however, significantly enhanced in infected labial and lung fibroblasts compared to foreskin-derived cells, and did not occur after infection with AD169. Together, these data indicate that fibroblast populations derived from different tissues are uniformly permissive to CMV infection but retain phenotypic differences of potential importance for infection-induced cell–cell fusion, and ensuing viral spread and pathogenesis in different organs.

## 1. Introduction

Horizontal transmission of human cytomegalovirus (CMV) is thought to occur by contact between contaminated bodily fluids such as urine and saliva and the epithelial layers of oronasal mucosae. Studies in mice and primates have indeed shown that infection of the oral mucosa leads to systemic dissemination and subsequent infection of multiple organs [1,2,3]. The earliest phases of CMV infection in humans, by contrast, have not been well investigated. Viralantigen- or DNA-positive cells have been detected in the alveoli and airways from patients with acute CMV infection or from lung transplant recipients [4,5], in lingual, laryngeal orpharyngeal ulcers [6,7,8,9,10,11,12,13,14], and in the gingival tissues of HIV+ individuals and/or periodontitis patients [15,16,17,18,19,20,21,22,23,24,25,26,27,28,29,30], suggesting that pulmonary, pharyngeal and oral epithelial cells are susceptible to infection and may, therefore, be targeted during entry. Ciliated respiratory epithelial cells were also recently reported to be permissive to infection, but the number of antigen-positive cells detected in nasal turbinates was low and viral yields were limited, suggesting that these cells might not be major ports of entry [31]. Therefore, either small numbers of initially infected nasal or oral epithelial cells are sufficient to mediate transmission, as suggested by some modeling efforts [32], or the routes whereby CMV is acquired during primary infection have not yet been found.

After some limited replication at surface sites, transmission of progeny virus to the underlying connective tissue fibroblasts or to vascular endothelial cells is thought to promote viral spread and dissemination within infected individuals [33].

In contrast to epithelial and endothelial cells, whose susceptibility to infection varies depending on their anatomical site of origin [4,34,35,36,37], fibroblasts from different tissues appear to consistently support the full viral replication cycle. This high degree of permissiveness may potentially amplify the viral loads in multiple organs such as the lungs, placenta, bone marrow, adrenal glands, heart, kidney, liver, spleen, stomach, colon, duodenum, pancreas, and small bowel [4,38,39,40], thus majorly contributing to viral pathogenesis in vivo.

Fibroblasts from foreskin, adenoid, uterine, and embryonic tissues have all been successfully used to isolate CMV, as found in clinical samples [41,42,43], with foreskin as well as MRC-5 fetal lung fibroblasts being the current standard cell types for propagating CMV to high titers [37,44]. The infection of gingival fibroblasts was proposed to contribute to the development of periodontitis in vivo by increasing the local production of proinflammatory cytokines and tissue remodeling enzymes [30,45,46], but no data currently exist regarding the ability of oral fibroblasts to support CMV replication. As infant labial fibroblasts may be involved in mother-to-child and child-to-child transmission via breast milk and saliva [47,48,49], we sought to evaluate the permissiveness to infection of fibroblasts isolated from the lips of a three-month-old infant.

We show that infant labial fibroblasts (LFs) are as permissive to CMV infection as foreskin fibroblasts (FFs) and MRC-5 fetal lung fibroblasts (MRs), indicating that oral fibroblasts may contribute to CMV acquisition and/or spread. Infection with the endothelial cell-adapted strain TB40/E [50] or with the epithelial cell-passaged stock TB40/EE [51] was also associated with the development of syncytia in significantly higher numbers in LF and MR cells compared to FF cultures, underscoring the existence of cell type-specific contributors to infection-induced cell–cell fusion. No syncytia were detected after infection with the attenuated strain AD169 [52], implying that viral factors, which were either exclusively or more abundantly expressed in the TB40/E-infected cells, are also involved. 

Together, these data suggest that, despite being similarly permissive to CMV replication, fibroblast populations from different organs possess distinct properties of potential relevance for CMV spread and pathogenesis within infected individuals, the manifestations of which are likely modulated by both viral and cellular factors. 

## 2. Materials and Methods

### 2.1. LF Isolation

Two small lip sections of approximately 0.5 cm × 0.5 cm × 0.3 cm in size, removed from a three-month-old male infant during cleft-lip repair surgery, were obtained from the Craniofacial Center at UCSF Benioff Children’s Hospital Oakland. Both fragments were incubated with the dermal side facing upwards in Dispase solution (CnT-DNP-10, CellnTec, Zen-Bio, Inc., Durham, NC, USA, final concentration: 2.4 U/mL) containing gentamycin/amphotericin (CnT-GAB10, CellnTec, final concentration 2×) for 17 h at 4 °C. The stromal layer was separated from the epithelium using curved forceps, and samples were incubated in Accutase^®^ solution (CnT-Accutase-100, CellnTec) at 37 °C for one hour, with gentle pipetting every ten minutes (min). The cell solution was then transferred into a 50 mL conical tube, while the tissue pieces were pressed onto a cell strainer and rinsed with abundant CnT-PR medium (CellnTec). Cells were centrifuged at 450× *g* for 10 min at room temperature (RT), resuspended in CnT-PR medium plus IsoBoost (CnT-ISO-50, CellnTec, final concentration 1.5×) and plated into a single well of a 24-well plate. At ten days post-isolation, cells had reached >90% confluency and were transferred by trypsinization into three wells of a 24-well plate in CnT-PR medium without IsoBoost or gentamycin/amphotericin (passage 1). Four days later, the medium was replaced with CnT-PR plus IsoBoost and gentamycin/amphotericin. Seventeen days post-transfer, the cells had reached 60–70% confluency and were transferred into a T25 flask in CnT-PR medium plus IsoBoost and gentamycin/amphotericin (passage 2). The supernatant was replaced with CnT-PR medium plus IsoBoost without gentamycin/amphotericin six and ten days later. Thirteen days post-transfer, the cells had reached 60–70% confluency and were transferred into a T75 flask in CnT-PR medium plus IsoBoost (passage 3). Four days after the third split, the cells had reached 60–70% confluency and were frozen in liquid nitrogen.

Mucosal cells from passage 3 were thawed and placed into one well of a six-well plate containing CnT-PR medium only. The medium was changed four days later and on day seven the cells were transferred into a T25 flask in CnT-PR medium only (passage 4). The medium was changed again seven and ten days later. 

Three weeks post-thawing, the first cells with clear spindle morphology started to appear. The medium was replaced with CnT-PR only on week four post-thawing, and as extensive cell death was noticed four days later, the cells were transferred again into one well of a six well plate in CnT-PR medium only (passage 5). Four days later, the medium was changed to Dulbecco’s modified Eagle medium (DMEM) and supplemented with 10% fetal clone serum III (HyClone), 100 U/mL penicillin, 100 μg/mL streptomycin, 4 mM HEPES, and 1 mM sodium pyruvate (Gibco, Life Technologies, New York, NY, USA) (DMEM complete medium) to encourage fibroblast growth. Three days post-medium change, the cells were transferred into a T25 flask (passage 6) and were then amplified in T175 flasks every 3–4 days until passage 12, when the cells were aliquoted and frozen in liquid nitrogen. 

### 2.2. Cell Culture and Infection

LFs, FFs (a gift from E. S. Mocarski), MRs (ATCC CCL-171), and ARPE-19 epithelial cells (ATCC^®^ CRL-2302) were propagated in DMEM complete medium. Cells at the indicated passage numbers were plated at a density of ~2 × 10^4^ cells/cm^2^ in 96-well plates or in 24-well plates containing glass coverslips three days before infection with CMV strain AD169varATCC [52] (a gift from E. S. Mocarski), TB40/E (a gift from C. Sinzger), or TB40/EE, an epithelial cell-passaged stock derived from TB40/E [51]. AD169 and TB40/E were propagated on FFs and concentrated by ultracentrifugation as previously described [53], while TB40/EE was collected from the supernatant of infected ARPE-19 cells. All virus stocks were titered on FFs, and the number of cells of each type present in the culture was counted prior to infection in order to accurately determine the amount of virus stock to use as an inoculum that would yield a multiplicity of infection (MOI) of 0.05, 0.5, or 1 plaque forming units (pfu)/cell. The virus inoculum was left in contact with the cells at 37 °C in 5% CO_2_ for four hours, after which the cells were washed twice and further incubated in complete DMEM. 

### 2.3. Immunofluorescence Staining

Cells grown on glass coverslips were fixed in 1.5% formaldehyde for 30 min at RT, permeabilized in 0.5% Triton-X 100 for 20 min on ice, treated with blocking buffer (20% fetal bovine serum in PBS) for 30 min at RT, and incubated with primary antibodies for one hour at RT in a humidified chamber. After washing in blocking buffer, the samples were incubated with primary antibodies, followed by Alexa Fluor 488- or 594-conjugated goat anti-mouse or goat anti-rabbit antibodies (1:200; Life technologies, Carlsbad, CA, USA) for one hour at RT. Nuclei were labeled with Hoechst 33,342 (0.2 mg/mL; Molecular Probes, Eugene, OR, USA) for three min at RT. Slides were then mounted in 90% glycerol–10% PBS containing 2.5 g/liter of 1, 4-diazabicyclo-(2, 2, 2)-octane (DABCO; Alfa Aesar, Pelham, NH, USA), and viewed on a Nikon Eclipse E600 fluorescence microscope equipped with an iVision-Mac imaging software. Antibodies used for staining were: mouse monoclonal antibody MAB810 (1:400, EMD Millipore, Burlington, MA, USA), which was directed against a common epitope to the viral IE1 and IE2 proteins (IE) [54], mouse anti-CMV pp28 (1:500, Virusys, clone CH19), rabbit-anti vimentin (1:100, Santa Cruz Biotechnology, Dallas, TX, USA, clone H-84), mouse anti-Pan-cytokeratin (1:100, Santa Cruz Biotechnology, clone AE13), mouse anti-cytokeratin 6, (1:100, Santa Cruz Biotechnology, clone B-7), rabbit anti-Von Willebrand Factor (1:100, Sigma, St. Louis, MO, USA, F3520), and rabbit anti-CD1a (1:100, Abcam, clone L21-A), rabbit anti-homeobox A11 (1:10, Sigma, HPA006770), and rabbit anti-homeobox B4 (1:300, Sigma, H0666).

### 2.4. Syncytia Counting

Micrographs of cell monolayers co-stained for IE and for pp28 were visually examined by at least two individuals for the presence of multi-nucleated cells sharing a single pp28+ virion assembly compartment (VAC). Each counted syncytium was delineated with a dashed line, and its shape (round, oval, irregular, etc.), inner diameter, outer diameter, area and nuclei content were recorded by each individual. Values were then compared and averaged if similar, or further assessed by a third individual if divergent. 

### 2.5. Reverse-Transcription Real-Time Quantitative PCR

Total RNA was extracted from FF, LF, MR, and ARPE-19 cells using the QIAGEN RNeasy Mini Kit (Qiagen, Hilden, Germany) and reverse transcribed with SuperScript III reverse transcriptase (Invitrogen, Life Sciences Solutions, Carlsbad, CA, USA). Real-time quantitative PCRs were performed in triplicate with PowerSYBR Green PCR Master Mix (Applied Biosystems, Life Technologies, Carlsbad, CA, USA) and an ABI7900 thermocycler (Applied Biosystems, Carlsbad, CA, USA) with primers hybridizing to the following cellular genes: homeobox A11 (HOXA11), forward: 5′-CGGCAGCAGAGGAGAAAG-3′, reverse: 5′-TATAGGGGCAGCGCTTTT-3′, homeobox B4 (HOXB4), forward: 5′-CCTGGATGCGCAAAGTTCA-3′, reverse: 5′-AATTCCTTCTCCAGCTCCAAGA-3′, homeobox B5 (HOXB5), forward: 5′-AATAGACGAGGCCAGCGCGT-3′, reverse: 5′-GGCCCGGTCATATCATGGCTGA-3′, homeobox B6 (HOXB6), forward: 5′-GTGCTCCACTCCGGTCTAC-3′, reverse: 5′-GTAACGTGTGTATGTCTGGCG-3′, THY1 forward: 5′-ATCGCTCTCCTGCTAACAGTC-3′, reverse: 5′-CTCGTACTGGATGGGTGAACT-3′ and GAPDH, used as a control, forward: 5′-TGCACCACCAACTGCTTAGC-3′, reverse: 5′-GGCATGGACTGTGGTCATGAG-3′. The following cycling parameters were used: 95 °C for 10 min to activate the AmpliTaq Gold DNA Polymerase, followed by 40 cycles of template denaturation at 95 °C for 15 s, and primer annealing and extension at 60 °C for 60 s. ΔCt values were used for relative quantification of gene expression vs. GAPDH, while 2^(ΔΔCt)^ values were used for fold change comparisons of gene expression levels in fibroblasts (FF, LF, or MR) vs. epithelial cells (ARPE-19).

### 2.6. Virus Titrations

The amount of cell-free virus that was present in the culture supernatants and the amount of cell-associated virus that was present in the cell pellets, which was released by sonication for ~three seconds using a Branson Ultrasonics Sonifier 150, were determined by infecting FFs with serial 10-fold dilutions of each sample, followed by cell staining at 24 hpi with MAb810 as described above.

## 3. Results

### 3.1. Labial Fibroblasts Are Permissive to CMV Replication

**Figure 1:**
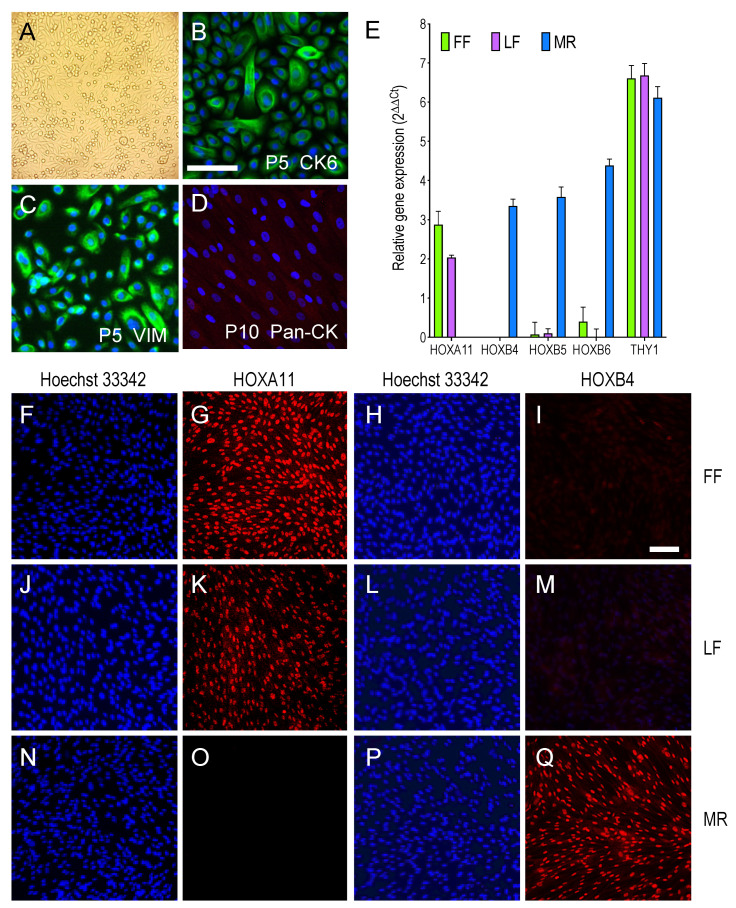
Cell morphology and expression of marker proteins in LF, FF, and MR cultures. (**A**) Representative light micrograph of passage zero oral mucosal cells at confluency (200× magnification). (**B**–**D**) Representative micrographs of passage five (P5) oral mucosal cells stained for cytokeratin 6 (CK6, green signal) or for vimentin (VIM, green signal), and of passage ten (P10) cells stained with anti-pan-cytokeratin (Pan-CK, red) antibodies. Hoechst 33,342 was used to highlight nuclei (blue). Identical signal patterns were observed in three replicate assays. (**E**) Relative expression (2^ΔΔCt^) of the homeobox A11, B4, B5, and B6, and THY1 genes in LF, FF, and MR cells as determined by reverse-transcription real-time quantitative PCR analysis. Mean and standard deviation values from replicate PCR wells in a single experiment are shown. (**F**–**Q**) Representative micrographs of LF (passage 18), FF (passage 25) and MR (passage 22) cells stained for HOXA11 or HOXB4 (red) and with Hoechst 33,342 (blue). Identical signal patterns were observed in two replicate assays. Bars = 100 μm.

A population of oral mucosal cells derived from the labial tissues of a three-month-old infant (Figure 1A) was cultured in CnT-PR medium, which supported the growth of epithelial cells until passage four, when the first cells with fibroblast-like morphology appeared. The culture medium was then switched to DMEM that was supplemented with 10% fetal clone serum III to encourage fibroblast expansion, leading to the complete supersedure of epithelial cells by passage seven. Although at passage five the cultures still retained the rounded, cobblestone-like morphology of epithelial cells and expressed keratin 6, which is a marker of labial epidermal cells [55] (Figure 1B), the mesenchymal cell marker vimentin [56] was already detectable in the majority of the cells (Figure 1C). Passage five cells also expressed keratin 4 and 5 at very low levels, and were uniformly negative for expression of the endothelial cell and megakaryocyte marker von Willebrand factor [57], and for the Langerhans-type dendritic cell marker CD1a [58] (not shown). 

From passage eight onwards all of the cells in the population displayed the characteristic fibroblast morphology and were vimentin-positive (not shown) but keratin-negative (Figure 1D). Akin to FF and MR, but not to ARPE-19 epithelial cells, LFs also transcribed the pan-fibroblast cell marker THY1/CD90 [59] (Figure 1E). To further characterize LF cell populations, the expression of genes known to be differentially transcribed in fetal lung (HOXB4, HOXB5, and HOXB6) vs. foreskin (HOXA11) fibroblasts [60,61] was evaluated. As expected, HOXB4, HOXB5, and HOXB6 were expressed in MR but not in FF cells, while HOXA11 was expressed in FF but not in MR cells, and LFs transcribed HOXA11 only (Figure 1E). Immunofluorescence staining of cultured LF (passage 18), FF (passage 25), and MR (passage 23) cells further confirmed that HOXA11 was exclusively present in FF and LF cells (Figure 1G,K), while HOXB4 was selectively found in MR cells (Figure 1Q). Therefore, and perhaps not surprisingly, LF cells appear to be closer to dermal-derived (e.g., FF) than lung-derived (e.g., MR) fibroblasts. Alternatively, these differences in HOX gene expression may be related to the developmental stage (fetal vs. infant/newborn) of the donors. When present, the signal from each protein was also observed in >99% of the nuclei in each population, suggesting that all cultures were highly homogeneous (Figure 1F–Q).

The ability of infant LF cells to support CMV replication was then compared to that of FF and MR cells. LF (passage 9 and 12), FF (passage 10 and 13), and MR (passage 21 and 22) cells grown in 24-well plates were infected with the fibroblast-adapted laboratory strain AD169 at an MOI of 0.05, 0.5, or 1 pfu/cell, or with the endothelial-cell-adapted strain TB40/E at an MOI of 1 pfu/cell. All virus titers were determined on FFs, which were used as a reference cell type. FF, MR, and LF cell numbers were also routinely counted prior to infection in order to accurately determine the amount of virus stock to use as the inoculum. The proportions of infected cells present at day one and two post-infection (pi) were then determined by staining for the viral immediate-early proteins IE1 and IE2 (IE), while the amounts of cell-associated and cell-free progeny present at days 3, 4, 5, and 6 pi were quantified by titration of cell sonicates and culture supernatants on FFs, respectively. 

**Figure 2:**
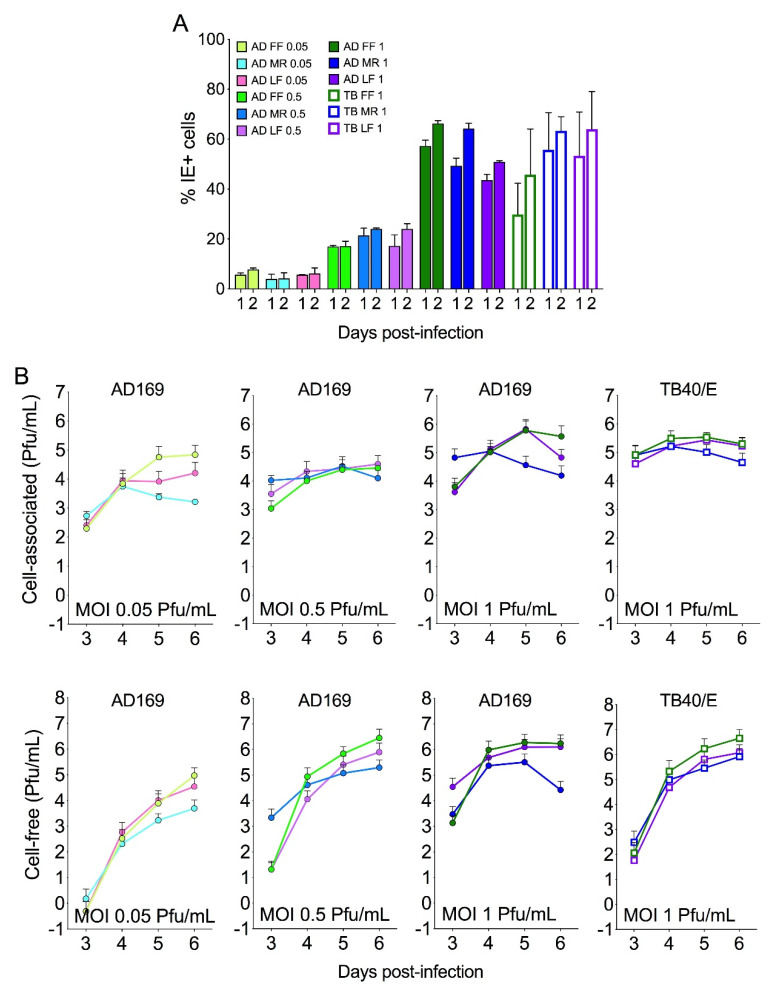
Infection and replication of CMV strains AD169 or TB40/E in LF, FF, or MR cultures. Confluent cultures in 24-well plates were exposed to AD169 (AD) at an MOI of 0.05, 0.5, or 1 pfu/cell, or to TB40/E (TB) at an MOI of 1 pfu/cell. Inoculum amounts were calculated based on the number of cells present in one well of each cell type at day zero (before infection). At each of the indicated days post-infection, cells and supernatants were collected and used to determine the proportion of nuclei expressing the IE1/IE2 proteins (% IE+, panel **A**) or the amounts of cell-free or cell-associated progeny produced by each culture, as titrated on FFs (panel **B**). Mean and standard deviation values from two (AD169) or four (TB40/E) separate experiments are shown.

No statistically significant differences were observed in the proportions of LF, FF, or MR cells supporting infection initiation after exposure to AD169 or TB40/E (Figure 2A), although, as previously observed [62], the percentage of IE + FF after infection with TB40/E was ~2-fold lower than after infection with AD169 at the same MOI (1 pfu/cell). These differences, however, were not statistically significant and appeared to be a characteristic unique to FF cultures. The amounts of cell-associated and cell-free progeny that were produced by LFs were also comparable to those produced by FFs and MRs after infection with AD169 at each MOI, or with TB40/E (Figure 2B). While MR cells displayed a tendency to produce less progeny than FF and LF cells at late (day 5–6) times post-infection with AD169, the cumulative frequency distributions of MR and FF titers were not significantly different according to the Kolmogorov-Smirnov test, and no differences were observed after infection with TB40/E. As previously reported [62], the amount of cell-free progeny released by TB40/E-infected cells at day three pi was 1–2 logs lower than that released by AD169-infected cells (Figure 2B). Together, these data show that, akin to FFs and MRs, LFs are fully permissive to CMV replication irrespective of the virus strain.

### 3.2. Syncytia Formation Is Enhanced in Labial and Lung Fibroblasts Compared to Foreskin Fibroblasts

**Figure 3:**
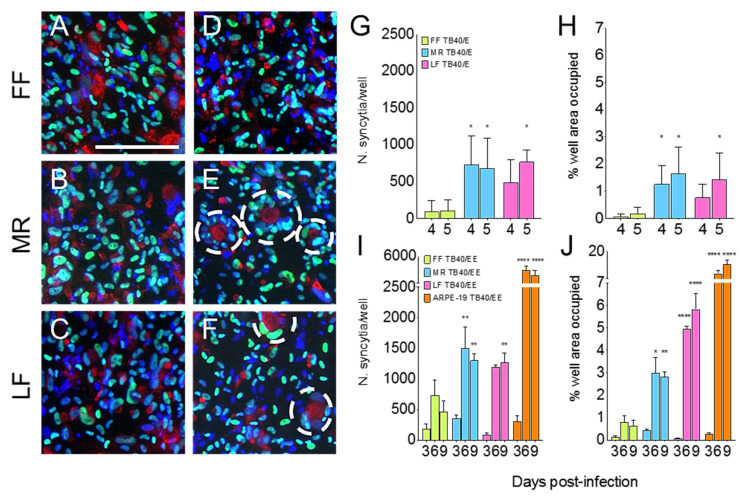
Syncytia formation in FF, MR, and LF cells infected with AD169, TB40/E, or TB40/EE. (**A**–**F**) Representative microscopy images of cells exposed to AD169 (**A**–**C**) or to TB40/E (**D**–**F**) virions at an MOI of 0.01 pfu/cell and stained for the viral IE1/IE2 proteins (green) and for the viral pp28 protein (red) at day nine post-infection. Dashed lines encircle syncytia. Bar = 200 μm. (**G**–**J**) Number (N.) of syncytia/well or calculated percentage of well area occupied by syncytia in fibroblast or ARPE-19 epithelial cell monolayers cultured in 24-well plates and exposed to TB40/E virions at an MOI of 0.5 (**G**,**H**) or TB40/EE virions at an MOI of 0.1 (**I**,**J**) pfu/cell. Mean and standard deviation values of counts obtained by two different individuals from at least four separate micrographs in a single experiment per strain are shown. * = *p* value > 0.01; ** = *p* value > 0.001; **** = *p* value < 0.0001 as determined by two-way ANOVA comparing LF or MR means to FF or ARPE-19 means.

Somewhat surprisingly, immunostaining of TB40/E-infected MR, and LF cell populations for the IE proteins and for the viral tegument protein pp28, the latter of which prominently accumulates in the virion assembly compartment (VAC) at late times pi, revealed the presence of syncytia (Figure 3E,F, dashed circles), whose size and abundance varied according to the cell type and virus strain (Figure 3G–J). 

While no heterokaryons were observed in AD169-infected cultures at any time pi (Figure 3A–C), the extent of cell–cell fusion occurring in FF monolayers after TB40/E infection was very limited, with most syncytia containing ~5 nuclei and having an area ~2.5-fold larger than that of a single fibroblast (estimated at 900 μm^2^ (Table 1). The calculated total number of syncytia per well was also small, so that overall, only ~0.1% of the well surface in the 24-well plates was occupied by fused FF cells (Figure 3H, green bars). By contrast, both MR and LF cultures contained significantly higher numbers of syncytia, which were also larger (~7 nuclei/syncytium, 4-fold larger than a single cell, Table 1), and hence occupied a 10-fold greater surface area than in the FF cultures (Figure 3H, azure and pink bars). 

We previously observed extensive syncytia formation in ARPE-19 retinal pigment epithelial cell populations infected with TB40/EE, which is an epithelial cell-passaged stock of TB40/E that, in contrast to TB40/E, can effectively enter and initiate infection in epithelial cells [51]. Passaging was associated with the selection of variants carrying an intact *UL128* open reading frame (ORF) [51,63], which presumably makes them capable of efficient entry into ARPE-19 cells due to the assembly of a functional gH/gL/UL128/UL130/UL131A pentameric complex (PC). As the PC is thought to contribute to syncytia formation [64,65], we sought to compare the syncytiogenic properties of epithelial and fibroblast cells by infecting ARPE-19, FF, MR, and LF cultures in parallel with TB40/EE at an MOI of 0.1 pfu/cell prior to immunostaining for IE and pp28 on days 3, 6, and 9 pi. Consistent with previous results [51], similar proportions of IE+ cells were observed in ARPE-19 (12 ± 4%), HF (13 ± 3%), MR (26 ± 9%), and LF (23 ± 3%) cells at day three pi, indicating that infection initiation was successful in all cell types. While the number and size of syncytia that were detected at day three pi were similar in all cell types, heterokaryons were again significantly larger and more abundant in LF and MR cells than in FF cultures (Table 1 and Figure 3F–G) occupying ~3% (MR, four-fold larger than FF) and ~5% (LF, eight-fold larger than FF) of the well surface on days six to nine pi. In addition, both the number of syncytia and the surface occupied by syncytia in ARPE-19 cell cultures were significantly (*p* < 0.001, two-way ANOVA) greater than in any fibroblast culture, suggesting that ARPE-19 epithelial cells are more prone to fusion after infection than fibroblasts. Although not directly compared, the size of the syncytia produced by TB40/E and TB40/EE in all three fibroblast types was very similar, while their number was ~3-fold higher in TB40/EE-infected cultures, suggesting that the ability to assemble a functional PC may support the initiation of cell–cell fusion in a larger proportion of cells infected with this stock. Finally, and importantly, syncytia formation was not observed at any time after the mock-infection of each culture, suggesting that FF, AR, and MR cells do not spontaneously fuse.

Together, these data indicate that the anatomical origin of fibroblasts does not significantly impact CMV replication but can modulate specific consequences of infection, such as the induction and magnitude of cell–cell fusion.

## 4. Discussion

On account of their ubiquitous distribution in the human body, and of their high permissiveness to CMV infection [4,40,66], fibroblasts are thought to play crucial roles in CMV transmission, spread, and pathogenesis. While oronasal epithelial cells are arguably the main entry targets of CMV that is present in breast milk, urine, and saliva, an infection of the fibroblasts comprising the deeper layers of the oral mucosa is likely needed to support local virus amplification and systemic dissemination following the transfer of viral progeny to the endothelial cells lining the dermal blood vessels. Similarly, the infection of oral fibroblasts with reactivated virus carried by extravasated myeloid cells is assumed to contribute to virus transmission by increasing viral loads in the saliva [67]. Oral fibroblasts have also been implicated in the etiology and/or aggravation of diseases such as gingivitis and periodontitis. The presence of CMV in the oral cavity of HIV+ individuals has been linked to the development of oral ulcers, acute necrotizing ulcerative gingivitis, and acute periodontal infections [15,16,17,18,19,20,21], while in HIV-negative individuals, CMV infection has been implicated in the onset and progression of severe periodontitis and of odontogenic cysts [22,23,24,25,26,27,28,29]. The detection of actively replicating CMV in the periodontal pockets of patients with juvenile or adult periodontitis [30] led to suggestions that fibroblast infection may lead to disease by inducing the release of tissue remodeling enzymes and proinflammatory cytokines in the extracellular environment [45,46], thereby increasing extracellular matrix destruction, reducing tissue integrity, diminishing protection against bacterial invasion [68], and/or triggering chronic inflammation [69,70]. Despite this, the ability of oral fibroblasts to support CMV replication has not been tested.

As labial tissues are prominently exposed to the external environment, and as CMV is usually acquired during infancy, we sought to evaluate the permissiveness to CMV infection of fibroblasts that were isolated from the lips of a less-than-three-month-old baby. We show that infant LFs are as permissive to CMV replication as the commonly used FF and MR cells, indicating that the anatomical site of origin has no impact on the ability of fibroblasts to support viral replication. While this is consistent with the detection of viral antigens in the mesenchymal cells of multiple organs (lungs, pancreas, bone marrow, colon, stomach, intestine, and placenta) from acutely infected individuals [4,38,39,40,71,72], it is in sharp contrast with data from epithelial and endothelial cells, whose permissiveness to infection is strongly affected by the site of origin. For instance, intestinal microvascular and aortic macrovascular endothelial cells are significantly more permissive than umbilical vein or brain microvascular endothelial cells [34,73], while aortic macrovascular or uterine microvascular endothelial cells produce much higher amounts of cell-free progeny than brain microvascular (~3-logs) [34], umbilical artery (1-log), lung microvascular (2-log), or umbilical vein endothelial cells (HUVEC, 2-log) [74]. Higher proportions of viral-antigen-expressing cells were also found in populations of retinal pigment (ARPE-19), cervical cancer (HeLa), non-small cell lung carcinoma (H1299), and breast cancer (MCF-7) epithelial cells (all >60%) compared to colon adenocarcinoma (SW480) or colorectal carcinoma (HCT116) epithelial cells (both <30%) [75]. At peak times, ARPE-19 cells also produced higher amounts of progeny compared to normal oral keratinocytes and telomerase-immortalized gingival cells [76]. 

As fibroblasts, epithelial, and endothelial cells are similarly heterogeneous [59,60,77], we speculate that the observed differences in susceptibility may depend on the expression of specific pro- or antiviral genes, with epithelial cell types that are directly exposed to the pathogen-rich environments of the nose, mouth, lungs, and vagina transcribing a wider set of anti-viral genes compared to the cells comprising the internal and more protected connective tissues. The involvement of fibroblasts in tissue nourishment, support, and repair also entails the synthesis of proteins involved in cell proliferation and energy metabolism, which may provide a more favorable environment for CMV replication. Genes encoding regulators of DNA/RNA synthesis, cell cycle progression, or mitochondrial energy production were indeed amongst the most transcriptionally induced in CMV-infected FFs [53].

The fact that fusion events were exclusively observed after infection with TB40/E or TB40/EE but not with AD169, and more readily occurred in LF and MR than in FF cultures, suggests that syncytia formation is both viral-strain- and fibroblast-type-dependent.

While AD169-infected cells may be resistant to fusion due to the presence of reduced amounts of gB (the viral fusogen), gH, gL, and/or gO, or to the synthesis of the “wrong” gB isoform [78], or to differences in gB, gH, gL, or gO glycosylation type or extent, we surmise that the inability to assemble a functional PC due to the presence of a frameshift mutation affecting *UL131A* [79] in the AD169 genome may be the primary culprit. Although addition of the UL128, UL130, and UL131A proteins did not appear to increase the extent of fusion in gB/gH/gL-expressing ARPE-19 cells [80], syncytia formation in ARPE-19 cultures that were infected by PC-positive CMV strains (TR-GFP, TB40-BAC4-GFP or VR1814), or in MR cultures that were infected by PC-positive variants (ABV or BAD*r*UL131-Y4), was effectively prevented in the presence of PC-specific antibodies [64,65,81]. Heterokaryons were also virtually non-existent in MR cells that were infected with PC-negative variants of CMV strains AD169 (HB15) or Towne (TS15) compared to infection with the carefully matched inocula of PC-positive variants of AD169 (BAD*r*UL131-Y4) or Towne (TS15-rN) [65]. Finally, we also observed lower numbers of syncytia following the infection with TB40/E compared to TB40/EE, which is a stock containing higher proportions of genomes carrying an intact *UL128* ORF [51,63], and is hence presumably better able to assemble functional PCs. Together, these data strongly suggest that the PC participates in syncytia formation, although the mechanisms involved remain unknown.

As antibodies to gB or gH/gL also interfere with syncytia formation in ABV- or BAD*r*-infected MR cells [65], the PC may be required to localize gB on the membrane of infected cells in sufficiently high amounts, or in the right conformation, in order to enable interactions with specific proteins on the membrane of neighboring cells. Alternatively, cellular proteins may interact with the PC itself. Of the two PC receptors identified thus far, olfactory receptor, family 14, subfamily I, member 1 (OR14I1) is not expressed in CMV-infected FFs or MRs [82,83,84], nor in donor-derived, uninfected fibroblasts [60], while neuropilin 2 (NRP2) is expressed at similarly low levels in CMV-infected FF and MR cells [83,84]. Therefore, neither appears to be a promising candidate.

For cells to merge, fusion-driving proteins must be present on one or both of the fusing membranes in order to bring the lipid bilayers into close proximity and allow for the opening of a fusion pore [85]. The same or different proteins can act on each membrane in homotypic or heterotypic bilateral fusions, respectively. Syncytia formation in infected fibroblasts may thus theoretically be driven by: (a) the same viral protein(s) or complex(es) present on both membranes, e.g., gB or the PC on both sides; (b) different viral protein(s) or complex(es) present on opposing membranes, e.g., gB on one side and gH/gL on the other, as observed in transduced ARPE-19 cells [80]; (c) the same cellular protein on both membranes (homotypic interactions); (d) different cellular proteins on each membrane (heterotypic interactions); or (e) viral protein(s) or complex(es) on one membrane and cellular receptor(s) on the other. Within this framework, the different propensities of FF, LF, and MR cells to form syncytia might be explained by the higher expression or membrane localization of viral or cellular “fusogens” in infected LF or MR cells compared to FFs, or by the absence of specific cellular receptors in FFs compared to MRs or LFs. As primary FFs were reported to be resistant to syncytia formation even after the forced expression of gB, gH, gL, and the gB ectodomain that was fused to the transmembrane and cytoplasmic domain of the VSV-G protein [80,86,87], we consider the absence of the appropriate cellular receptors to be the most likely culprit. Interestingly, syncytia formation was consistently reported to occur not only in MR cells [65,78,88,89], but also in WI-38 [90] and other “human embryo lung” fibroblast lines [91,92,93], suggesting that a stronger tendency to fuse may be an intrinsic property of lung fibroblasts. While this may simply depend, again, on viral protein expression levels, the selective expression of *cellular* surface receptors and/or regulators of membrane or cytoskeleton dynamics in MR cells is likely to contribute. 

In summary, while the universal ability of fibroblasts to support CMV infection may suggest that organotypic properties are unlikely to be relevant to CMV pathogenesis in vivo, our data show that fibroblasts derived from different organs have significantly different properties, some of which, such as the ability to support cell–cell fusion at late times pi, may be quite significant for CMV spread, transmission, and escape from antibody detection, with direct effects on the infection-associated disease and vaccine efficacy.

## Figures and Tables

**Table 1:** Nuclei content and calculated area of syncytia present in monolayers of FF, MR, LF, or ARPE-19 cells infected with TB40/E (MOI of 0.5 pfu/cell) or TB40/EE (MOI of 0.1 pfu/cell). Mean and standard deviation values are reported.

Cell Type	CMV Stock	Day pi	N. Counted	N. Nuclei	Area (μm^2^)
FF	TB40/E	4	2	4 ± 1	1204 ± 657
		5	3	4 ± 2	2902 ± 2310
MR	TB40/E	4	8	6 ± 2	3089 ± 516
		5	5	8 ± 4	4327 ± 3305
LF	TB40/E	4	6	6 ± 2	2829 ± 1740
		5	7	8 ± 4	4428 ± 4123
FF	TB40/EE	3	8	3 ± 1	1277 ± 553
		6	11	4 ± 2	1960 ± 1022
		9	11	5 ± 2	2426 ± 2279
MR	TB40/EE	3	15	4 ± 2	2181 ± 1387
		6	14	9 ± 7	3569 ± 2206
		9	12	10 ± 5	3903 ± 2589
LF	TB40/EE	3	5	2 ± 0.4	1280 ± 158
		6	11	10 ± 4	7452 ± 6577
		9	14	11 ± 5	8294 ± 4959
ARPE-19	TB40/EE	3	9	4 ± 2	1484 ± 1265
		6	13	12 ± 8	4201 ± 3500
		9	14	19 ± 9	6576 ± 5182
